# Novel Antidiabetic Drugs and Risk of Venous Thromboembolism: A Literature Review

**DOI:** 10.1055/a-2546-0353

**Published:** 2025-03-28

**Authors:** Qingui Chen, Rayna J.S. Anijs, Judith P.L. Verlaan, Luuk J.J. Scheres, Frederikus A. Klok, Suzanne C. Cannegieter

**Affiliations:** 1 Department of Clinical Epidemiology, Leiden University Medical Center, Leiden, The Netherlands; 2 Department of Medicine, Thrombosis and Hemostasis, Leiden University Medical Center, Leiden, The Netherlands; 3 The Knowledge Institute of the Federation of Medical Specialists, Utrecht, The Netherlands; 4 Department of Internal Medicine, Radboud University Medical Center, Nijmegen, The Netherlands

**Keywords:** glucagon-like peptide-1 receptor agonists, dipeptidyl-peptidase IV inhibitors, sodium-glucose transporter 2 inhibitors, venous thromboembolism, hypoglycemic agents

## Abstract

Novel antidiabetic drugs, particularly sodium-glucose cotransporter 2 (SGLT2) inhibitors and glucagon-like peptide-1 (GLP-1) receptor agonists, have significantly transformed the management landscape for type 2 diabetes mellitus, cardiovascular diseases, and chronic kidney diseases, owing to their well-established cardiorenal protective effects. Given the shared risk factors and comorbidities, it is relevant to consider the potential risk of venous thromboembolism (VTE) in individuals prescribed these novel antidiabetic medications. This literature review aims to summarize currently available evidence on VTE risk associated with novel antidiabetic drugs, including GLP-1 receptor agonists, dipeptidyl-peptidase IV (DPP-4) inhibitors, and SGLT2 inhibitors. Following a comprehensive search on PubMed using relevant keywords and backward reference searching, we identified 25 publications that directly reported on associations between these medications and VTE risk. Findings from these studies, including seven meta-analyses, reveal inconsistent results: some studies suggest that GLP-1 receptor agonists or DPP-4 inhibitors may be associated with increased risk of VTE, whereas SGLT2 inhibitors do not appear to be associated with VTE and may even be a protective factor. A notable limitation of the existing studies is the significant challenge posed by confounding in observational studies, while the randomized controlled trials (RCTs) often concluded with a limited number of VTE events, if it was studied. Furthermore, all identified studies focused on the risk of primary VTE, leaving an important knowledge gap regarding whether these novel antidiabetic drugs may influence the efficacy or safety of anticoagulants used for preventing VTE recurrence. Addressing these gaps presents an important avenue for future research.

Several new classes of antidiabetic drugs have been approved since 2005, 1 2 including glucagon-like peptide-1 (GLP-1) receptor agonists, dipeptidyl-peptidase IV (DPP-4) inhibitors, and sodium-glucose cotransporter 2 (SGLT2) inhibitors. Although these agents were initially approved for glucose control or weight reduction, specifically in patients with type 2 diabetes mellitus (T2DM), over the past decade, the additional cardiovascular and renal protective effects of SGLT2 inhibitors and GLP-1 receptor agonists have become increasingly evident. 3 4 5 6 Furthermore, these benefits have been validated in patients without T2DM, including those with heart failure, chronic kidney disease, or obesity (with preexisting cardiovascular disease). 7 8 9 These developments have revolutionized the management of T2DM, 10 11 cardiovascular diseases, 12 13 14 chronic kidney diseases, 15 and obesity, 16 as evidenced by a marked increase in the usage of these medications in recent years. 17 18 A similar increase has been observed in the use of DPP-4 inhibitors among T2DM patients, 19 despite their neutral cardiovascular and renal effects. 10

Unlike the well-documented effects of these novel antidiabetic drugs on arterial thromboembolic complications, such as ischemic stroke and myocardial infarction, 20 21 the potential impact of these agents on the occurrence of venous thromboembolism (VTE) remains unclear. Most of the clinical indications for these drugs, including diabetes, 22 heart failure, 23 chronic kidney disease, 24 and obesity, 25 are associated with shared risk factors for VTE or may even directly contribute to VTE development, and they are as well as linked to risk factors for VTE recurrence. 26 27 28 29 These conditions are also highly prevalent among patients with VTE or those at increased risk of VTE. The aim of this review is to summarize the available literature focusing on the risk of VTE in individuals receiving these novel antidiabetic drugs, thereby providing insights for future research directions.

## Methods

We conducted a literature search on PubMed covering publications from inception up to February 5, 2025. The keywords used in the search included GLP-1 receptor agonists, DPP-4 inhibitors, SGLT2 inhibitors, and VTE. The detailed search strategy is provided in Appendix A1 (available in the online version only). No language restrictions were applied. The initial search yielded 46 results, which were subsequently reviewed for inclusion.

**Table 1:** Summary of available evidence

Study	Publication year	Study period	Study design	Study population	Main findings
GLP-1RA					
Yin et al. 31	2021	Up to October 2020	Meta-analysis (21 RCTs?) GLP-1RA (semaglutide) versus controls	T2DM patients	Semaglutide significantly increased the risk of DVT (RR 3.66, 1.09–12.25) but not with PE (RR 0.97, 0.47–1.99)
Liao et al. 32	2022	Up to March 2021	Meta-analysis (6 RCTs) GLP-1RA (lixisenatide, albiglutide, liraglutide, semaglutide, dulaglutide) versus placebo	T2DM patients	GLP-1RA was associated with a higher risk of DVT (RR 2.12, 1.32–3.40)
Almeida et al. 33	2024	Up to November 2023	Meta-analysis (4 RCTs) GLP-1RA (liraglutide, semaglutide) versus placebo	Patients with obesity or overweight	GLP-1RA was not associated with DVT (RR 0.30, 0.06–1.40)
Elsabbagh et al. 44	2024	2010–2022	Cohort study GLP-1RA (dulaglutide, exenatide, liraglutide, lixisenatide, semaglutide; *n* = 5,010) versus non-users ( *n* = 18,701)	T2DM patients undergoing total shoulder arthroplasty	No significant increase in 90-day major complications, including DVT (OR 1.27, 0.82–1.91) and PE (OR 0.70, 0.40–1.26)
Seddio et al. 45	2024	2010–2022	Cohort study GLP-1RA (semaglutide, *n* = 1,094) versus non-users ( *n* = 4,110)	T2DM patients undergoing primary arthroscopic rotator cuff repair	Semaglutide was associated with a lower risk of VTE within 90 days after the surgery (OR 3.10, 1.85–5.64; non-users vs. semaglutide)
Wang et al. 34	2024	Up to August 2023	Meta-analysis (11 RCTs) GLP-1RA (efpeglenatide, lixisenatide, albiglutide, liraglutide, semaglutide, dulaglutide, tirzepatide) versus controls	T2DM patients	GLP-1RA was associated with a higher risk of DVT (RR 1.92, 1.23–3.00)
Lawand et al. 46	2024	2012–2023	Cohort study GLP-1RA (semaglutide or liraglutide, *n* = 1,259) versus non-users ( *n* = 1,259)	Patients undergoing primary total shoulder arthroplasty	GLP-1RA was associated with a higher risk of DVT within 90 days after the surgery (OR 3.0, 1.5–5.9) but not with PE (OR 1.6, 0.9–3.1)
Kim et al. 47	2024	2010–2022	Cohort study GLP-1RA (exenatide, semaglutide, dulaglutide, or liraglutide, *n* = 2,975) versus non-users ( *n* = 2,975)	Morbidly obese patients undergoing primary total knee arthroplasty	GLP-1RA was not associated with DVT within 90 days after the surgery (1.0% vs. 1.1%) but a lower risk of PE (<0.4% vs. 0.6%, *p* = 0.050)
DPP-4i					
Arnaud et al. 41	2017	2005–2015	An automated system combining safety signal detection and prioritization from health care databases	A French health care database	There was a strong new signal with DPP-4i and VTE
Gouverneur et al. 42	2020	October 2006–December 2019	Disproportionality analyses	WHO global database of spontaneous reporting adverse drug reactions	There was an excess of reporting of VTE with DPP-4i compared with other non-insulin glucose-lowering drugs, but only for sitagliptin
Lu et al. 43	2020	2004–2018	Disproportionality analyses DPP-4i versus other glucose-lowering drugs, including SGLT2i	FDA adverse event reporting system	No association between DPP-4i and VTE Moderate to strong signals of portal vein thrombosis, splenic vein thrombosis, and mesenteric vein thrombosis risks
Xin et al. 35	2021	Up to May 2020	Meta-analysis (5 RCTs) DPP-4i (saxagliptin, alogliptin, sitagliptin, omarigliptin, linagliptin) versus placebo	T2DM patients	DPP-4i was not associated with VTE (OR 1.12, 0.81–1.55) or PE (OR 1.14, 0.68–1.90)
Deischinger et al. 48	2022	1997–2014	Case–control study	T2DM patients	DPP-4i was associated with VTE in female T2DM patients (OR 2.3, *p* = 0.0096) but not among males (OR 1.6, *p* = 0.28)
Liao et al. 32	2022	Up to March 2021	Meta-analysis (5 RCTs) DPP-4i (linagliptin, alogliptin, omarigliptin, saxagliptin, sitagliptin) versus placebo	T2DM patients	DPP-4i was not associated with DVT (RR 0.92, 0.54–1.57)
SGLT2i					
Wang et al. 36	2019	Up to April 2019	Meta-analysis (29 RCTs) SGLT2i (canagliflozin, dapagliflozin, empagliflozin, ertugliflozin) versus controls	T2DM patients	SGLT2i was not associated with VTE (RR 0.98, 0.75–1.28), DVT (RR 1.06, 0.60–1.89), or PE (0.99, 0.67–1.46).
Patel et al. 38	2020	–	Pooled analysis (7 RCTs) SGLT2i (ertugliflozin) versus controls	T2DM patients	SGLT2i was not associated with VTE
Liao et al. 32	2022	Up to March 2021	Meta-analysis (9 RCTs) SGLT2i (canagliflozin, dapagliflozin, empagliflozin, ertugliflozin) versus placebo	Patients with T2DM (6 RCTs), heart failure (2 RCTs), or chronic kidney disease (1 RCT)	SGLT2i was not associated with DVT (RR 1.03, 0.69–1.53)
Stöllberger et al. 39	2023	–	Systematic review	Congestive heart failure patients	Only two trials 121 130 reported VTE as adverse events, but the incidence was low and did not differ between SGLT2i and placebo
Wang et al. 34	2024	Up to August 2023	Meta-analysis (14 RCTs) SGLT2i (canagliflozin, dapagliflozin, empagliflozin, sotagliflozin) versus placebo	Patients with T2DM, heart failure, or chronic kidney disease	SGLT2i was not associated with DVT (RR 0.80, 0.58–1.11)
SGLT2i versus GLP-1RA					
Ueda et al. 49 (Caparrotta et al. 40 ) a	2018 (2021)	July 2013–December 2016	Cohort study (systematic review) SGLT2i (dapagliflozin, empagliflozin, canagliflozin, *n* = 17,213) versus GLP-1RA (liraglutide? *n* = 17,213)	T2DM patients	No difference in VTE (HR 0.99, 0.71–1.38)
Patil et al. 50	2023	April 2013–September 2020	Cohort study SGLT2i (empagliflozin, canagliflozin, dapagliflozin; *n* = 35,347) versus GLP-1RA (liraglutide, dulaglutide, lixisenatide, orsemaglutide; *n* = 35,347)	T2DM patients	SGLT2i was not associated with VTE (HR 1.02, 0.80–1.30)
SGLT2i versus DPP-4i					
Schmedt et al. 51	2021	2012–2018	Case–control study? SGLT2i ( *n* = 4,705) versus DPP-4i ( *n* = 14,840)	T2DM patients	SGLT2i was associated with a lower VTE risk (RR 0.75, 0.59–0.94) for both dapagliflozin and empagliflozin
Aloe et al. 52	2023	2013–2017	Cohort study SGLT2i ( *n* = 5,414) versus DPP-4i ( *n* = 5,414)	T2DM patients	SGLT2i was not associated with VTE (HR 0.65, 0.34–1.25) among both prevalent and incident users
Tsai et al. 53	2024	May 2016–December 2020	Cohort study SGLT2i ( *n* = 123,567) versus DPP4-i ( *n* = 578,665)	T2DM patients	SGLT2i was associated with a lower risk of retinal vein occlusion (HR 0.76, 0.59–0.98), but for branch retinal vein occlusion only
DPP-4i, SGLT2i, and GLP-1RA					
Tsai et al. 37	2024	May 2016–December 2020	Cohort study DPP-4i ( *n* = 598,280), SGLT2i ( *n* = 136,530), versus GLP-1RA ( *n* = 5,760)	T2DM patients	SGLT2i versus DPP-4i was associated with a lower VTE risk (HR 0.70, 0.59–0.84) but not with GLP-1RA (HR 1.39, 0.32–5.94)
		Up to May 2023	Meta-analysis (3 observational studies)	T2DM patients	SGLT2i versus DPP-4i: lower VTE risk (HR 0.71, 0.62–0.82); SGLT2i versus GLP-1RA: no association (HR 0.91, 0.73–1.15)

Abbreviations: DPP-4i, dipeptidyl-peptidase IV inhibitor; DVT, deep vein thrombosis; FDA, Food and Drug Administration; GLP-1RA, glucagon-like peptide-1 receptor agonist; HR, hazard ratio; OR, odds ratio; PE, pulmonary embolism; RCT, randomized controlled trial; RR, risk ratio; SGLT2i, sodium-glucose transporter 2 inhibitor; T2DM, type 2 diabetes mellitus; VTE, venous thromboembolism; WHO, World Health Organization.

a A systematic review referring to the study by Ueda et al. 49

The primary inclusion criterion was human studies, encompassing observational studies, RCTs (or secondary analyses of RCTs), and meta-analyses that reported on the risk of VTE, including deep vein thrombosis (DVT), pulmonary embolism (PE), or both, in patients receiving one of the three classes of novel antidiabetic drugs compared with those receiving placebos or other antidiabetic drugs (including other classes of novel antidiabetic drugs). Studies that assessed associations between novel antidiabetic drugs and VTE using methods such as disproportionality analyses 30 were also included. General reviews, case reports, comments, or fundamental research were generally excluded; however, given the limited number of relevant publications, the full text of all identified publications was reviewed, and specific sections were carefully examined where appropriate and necessary. Additionally, relevant studies identified through reference lists of included publications were considered for inclusion. Since RCTs on novel antidiabetic drugs often included VTE as a safety outcome but ultimately observed very few events, we did not further review details of these RCTs (unless they were captured in our literature search). Instead, meta-analyses of these RCTs (if any) were reviewed.

For each identified study, we extracted information such as publication year, study period, study design, study population, and main findings. Information from other publications relevant to the review topic, such as potential mechanisms underlying the associations between novel antidiabetic drugs and VTE, may be mentioned in the discussion, though these references are not exhaustive.

## Results

As shown in Table 1 , 25 studies (including 1 identified through backward reference searching) were ultimately included. These comprised 7 meta-analyses, 31 32 33 34 35 36 37 1 pooled analysis of RCTs, 38 2 systematic reviews, 39 40 and 3 disproportionality analyses, 41 42 43 in addition to 11 traditional observational studies. 37 44 45 46 47 48 49 50 51 52 53

### Glucagon-like Peptide-1 Receptor Agonists and Venous Thromboembolism Risk

A total of eight studies 31 32 33 34 44 45 46 47 were identified that compared VTE risks between individuals receiving GLP-1 receptor agonists and those not receiving these agents, with inconsistent results reported. A meta-analysis by Yin et al. 31 included 21 RCTs (i.e., the PIONEER 54 55 56 57 58 59 60 61 62 63 and SUSTAIN trials 64 65 66 67 68 69 70 71 72 73 74 ) designed to evaluate the efficacy of semaglutide in T2DM patients. Among the various investigated safety outcomes, semaglutide was associated with an increased risk of DVT (8/5,844 vs. 1/7,495; risk ratio [RR] 3.66, 95% confidence interval [CI] 1.09–12.25) but not with PE (11/5,303 vs. 19/7,565; RR 0.97, 95% CI 0.47–1.99). However, the trials included for the analyses of DVT or PE are not reported. Another meta-analysis by Liao et al., 32 which included six RCTs involving T2DM patients, 5 55 64 75 76 77 consistently found that GLP-1 receptor agonists (various subtypes vs. placebo) were associated with an increased risk of DVT (55/20,598 vs. 25/20,608; RR 2.12, 95% CI 1.32–3.40). Wang et al. 34 conducted a meta-analysis including 11 RCTs involving T2DM patients 5 54 55 64 67 75 76 77 78 79 80 (various subtypes, mostly with placebo as the control arm, but also including two trials using DPP-4 inhibitors and one using insulin) and similarly reported an increased DVT risk (62/29,005 vs. 28/25,078; RR 1.92, 95% CI 1.23–3.00). In contrast, another meta-analysis 33 found no association between GLP-1 receptor agonists (including liraglutide and semaglutide) and DVT risk (2/5,015 vs. 3/2,237; RR 0.30, 95% CI 0.06–1.40), although this study focused on patients with obesity or overweight conditions. 81 82 83 84

Additionally, four cohort studies examined postoperative VTE risk in T2DM or morbidly obese patients receiving GLP-1 receptor agonists. 44 45 46 47 The study by Elsabbagh et al. 44 investigated DVT or PE risk within 90 days after total shoulder arthroplasty and found no significant difference in DVT risk (odds ratio [OR] 1.27, 95% CI 0.82–1.91) or PE risk (OR 0.70, 95% CI 0.40–1.26) between GLP-1 receptor agonist users and non-users. Conversely, Lawand et al. 46 found those who received GLP-1 receptor agonists after the same procedure had a significantly higher DVT risk (OR 3.0, 95% CI 1.5–5.9), although there was no significant difference in PE risk (OR 1.6, 95% CI 0.9–3.1). The studies by Seddio et al. 45 and Kim et al. 47 examined VTE risk after two other procedures (i.e., arthroscopic rotator cuff repair and total knee arthroplasty), and both found GLP-1 receptor agonists were a protective factor against VTE (or PE) within 90 days after the procedures. These observational studies often employed matching methods (propensity-score matching or exact matching) to control for confounding factors. Only the study by Kim et al. 47 clearly reported postoperative prophylactic anticoagulation, which might not be routinely provided in the other three studies 44 45 46 (according to the types of procedure).

### Dipeptidyl-peptidase-IV Inhibitors and Venous Thromboembolism Risk

A total of six studies 32 35 41 42 43 48 examined the association between DPP-4 inhibitors and VTE risk. Three of these studies used public health care data to detect drug safety signals, with two identifying a VTE safety signal associated with DPP-4 inhibitors, 41 42 while one did not find such an association. 43 In detail, Arnaud et al. 41 analyzed data from a French health care database and found safety signals indicating associations between PE and vidagliptin, PE and saxagliptin, and VTE and sitagliptin. Gouverneur et al. 42 used the World Health Organization global database of individual case safety reports of potential adverse drug reactions and performed disproportionality analyses, finding an excess of VTE reports associated with DPP-4 inhibitors compared with other non-insulin glucose-lowering drugs, though the excess risk appeared to be limited to sitagliptin users based on a post hoc subgroup analysis. However, Lu et al., 43 using data from spontaneous reporting systems of drug adverse events, conducted disproportionality analyses and found no signal of an association between DPP-4 inhibitors and VTE risk, though there were moderate to strong signals for risks of portal vein thrombosis, splenic vein thrombosis, and mesenteric vein thrombosis.

Two meta-analyses 32 35 consistently reported no association between DPP-4 inhibitors and VTE in T2DM patients. The meta-analysis by Xin et al., 35 which included five cardiovascular outcomes trials, 85 86 87 88 89 found no association between DPP-4 inhibitors (various subtypes vs. placebo) and VTE (79/23,899 vs. 70/23,815; OR 1.12, 95% CI 0.81–1.55) or PE (31/23,899 vs. 27/23,815; OR 1.14, 95% CI 0.68–1.90). Similarly, Liao et al. 32 included five RCTs 85 86 87 88 89 and found no association with DVT risk (26/23,833 vs. 28/23,750; RR 0.92, 95% CI 0.54–1.57). In contrast, a case–control study reported that DPP-4 inhibitors were associated with increased VTE risk in female T2DM patients only (OR 2.3, *p* = 0.0096). 48 However, details regarding confounding control were not clearly provided, as this was an additional analysis within the study.

### Sodium-Glucose Cotransporter 2 Inhibitors and Venous Thromboembolism Risk

Five studies examined VTE risk in patients receiving SGLT2 inhibitors, including three meta-analyses, 32 34 36 a pooled analysis of RCTs, 38 and a systematic review. 39 These studies consistently reported no association between SGLT2 inhibitors and VTE or DVT. Wang et al. 36 performed a meta-analysis of 29 RCTs 3 90 91 92 93 94 95 96 97 98 99 100 101 102 103 104 105 106 107 108 109 110 111 112 113 114 115 116 117 and found no significant difference in VTE risk between T2DM patients receiving SGLT2 inhibitors and those who did not (128/32,038 vs. 92/23,997; RR 0.98, 95% CI 0.75–1.28), with consistent results for DVT and PE separately. Most of the control arms in these RCTs were placebo, although some used other antidiabetic drugs, including DPP-4 inhibitors. Liao et al. 32 included nine RCTs 3 7 8 113 115 117 118 119 (note that trials NCT01032629 and NCT01989754 are in one publication 113 ) in their meta-analysis, reporting no association (vs. placebo) between SGLT2 inhibitors and DVT risk (61/33,124 vs. 48/26,568; RR 1.03, 95% CI 0.69–1.53). Although the majority of the included RCTs involved T2DM patients, two studies included heart failure patients, 7 118 and one involved chronic kidney disease patients. 8 Another meta-analysis by Wang et al. 34 included 14 RCTs 3 7 8 113 115 117 118 119 120 121 122 123 124 and similarly found no association between SGLT2 inhibitors (vs. placebo) and DVT risk (80/48,446 vs. 86/41,886; RR 0.80, 95% CI 0.58–1.11), with trials involving patients with T2DM, heart failure, or chronic kidney disease.

In addition to these three meta-analyses, a pooled analysis 38 of seven RCTs 112 114 125 126 127 128 129 reported no association between SGLT2 inhibitors and VTE risk in T2DM patients, though limited details were provided regarding this outcome. A systematic review 39 identified only two trials 121 130 involving heart failure patients that reported VTE as an adverse event, with a low incidence that did not differ between SGLT2 inhibitors and placebo.

### Venous Thromboembolism Risk among the Three Classes of Novel Antidiabetic Drugs

The remaining studies primarily compared VTE risk among the three classes of novel antidiabetic drugs ( Table 1 ), although no studies were specifically identified that directly compared GLP-1 receptor agonists with DPP-4 inhibitors.

Two cohort studies, both involving T2DM patients, compared patients receiving SGLT2 inhibitors to those receiving GLP-1 receptor agonists and found no difference in VTE risk. 49 50 Ueda et al. 49 utilized nationwide data from Sweden and Denmark, including 17,213 patients on SGLT2 inhibitors and GLP-1 receptor agonists, respectively, with a median follow-up of about 9 months, found no association between SGLT2 inhibitors and VTE (hazard ratio [HR] 0.99, 95% CI 0.71–1.38; absolute risk difference 0.2, 95% CI −0.4 to 1.3). Confounding was controlled using an active comparator new-user study design and propensity-score matching. Similarly, Patil et al. 50 conducted a study using nationwide U.S. data, with an approximately doubled sample size ( *n* = 35,347) and slightly longer median follow-ups (i.e., 1.01 and 1.54 years, respectively), and reported that SGLT2 inhibitors were not associated with VTE risk (HR 1.02, 95% CI 0.80–1.30) compared with GLP-1 receptor agonists.

Three observational studies compared VTE risk between SGLT2 inhibitors and DPP-4 inhibitors in T2DM patients. 51 52 53 Schmedt et al. 51 used a nested case–control design to include 2,152 VTE cases and 85,104 matched controls, finding that SGLT2 inhibitors were associated with a lower VTE risk compared with DPP-4 inhibitors (RR 0.75, 95% CI 0.59–0.94, though the estimate may actually represent OR based on the described methodology). This association was consistently observed by subtypes of SGLT2 inhibitors (i.e., dapagliflozin or empagliflozin). Similar results were observed in a cohort study, 53 which found that SGLT2 inhibitors were associated with a reduced risk of retinal vein occlusion compared with DPP-4 inhibitors (HR 0.76, 95% CI 0.59–0.98), though this association was only observed for branch retinal vein occlusion and not central retinal vein occlusion. Conversely, Aloe et al. 52 found no significant reduction in VTE risk with SGLT2 inhibitors compared with DPP-4 inhibitors ( *n* = 5,414 each arm; HR 0.65, 95% CI 0.34–1.25), with consistent results in both prevalent and incident users.

Tsai et al. 37 investigated potential differences in VTE risk between SGLT2 inhibitors and GLP-1 receptor agonists, as well as between SGLT2 inhibitors and DPP-4 inhibitors, using the same dataset. Their findings aligned with previous studies, indicating no association between SGLT2 inhibitors and VTE risk (HR 1.39, 95% CI 0.32–5.94) compared with GLP-1 receptor agonists in T2DM patients, but a lower VTE risk was observed compared with DPP-4 inhibitors (HR 0.70, 95% CI 0.59–0.84). In the same study, the authors performed a meta-analysis that combined their data with three other observational studies, 49 51 52 and the results remained unchanged.

## Discussion

This review examined the associations of novel antidiabetic drugs, including GLP-1 receptor agonists, DPP-4 inhibitors, and SGLT2 inhibitors, with the risk of VTE. The review confirmed the scarcity of data on this topic, as only 25 relevant studies were identified. Although seven of these studies were meta-analyses of RCTs, the total number of VTE events included across these RCTs was very limited. This limitation likely arises because the sample sizes of such RCTs are often determined based on primary efficacy outcomes, making them underpowered to detect effects on rare safety outcomes like VTE, especially given the relatively short follow-up periods. Furthermore, recent RCTs involving GLP-1 receptor agonists and SGLT2 inhibitors included populations with or at high risk of cardiovascular diseases, who may already be receiving antithrombotic therapy (e.g., ∼60% 117 ), thereby further reducing the absolute risk of VTE. Nevertheless, future RCTs are still encouraged to include VTE, including both DVT and PE, as a secondary outcome. Considering the potential effect modification by subtype of the same drug class or by patient characteristics, as suggested by some studies, 42 48 more research, both RCTs and observational studies, is undoubtedly needed.

This review also revealed significant inconsistency in the available evidence, with some studies indicating that GLP-1 receptor agonists or DPP-4 inhibitors may be linked to an increased risk of VTE, while SGLT2 inhibitors do not appear to be associated with VTE and may even have a protective effect. Based on the current evidence (albeit still limited), it may be reasonable to infer that SGLT2 inhibitors could be preferred in the context of VTE risk, whereas it remains unclear whether GLP-1 receptor agonists or DPP-4 inhibitors influence VTE risk. Nevertheless, a statistically significant difference in VTE risk on a relative scale may still be clinically irrelevant, while data on the difference in VTE on an absolute scale are rarely reported. Given their expanding indications 10 11 12 13 14 15 16 and the health care burden related to VTE, 131 determining the effects of all these novel antidiabetic drugs on VTE risk is of great clinical importance, especially as these medications, particularly SGLT2 inhibitors and GLP-1 receptor agonists, are increasingly being prescribed to patients for whom VTE risk is a consideration.

Obtaining high-quality evidence regarding the association of novel antidiabetic drugs with VTE risk is challenging. Given the low absolute incidence of VTE at the population level, conducting an RCT with sufficient statistical power to evaluate the effect of these drugs on VTE could be impractical. Observational studies utilizing routinely collected health care data might be a more feasible and even preferable approach, as their findings are more generalizable to everyday clinical practice. Nonetheless, there are challenges to this approach. First, these medications, particularly SGLT2 inhibitors and GLP-1 receptor agonists, are relatively new, and accumulating large-scale data on their use will require time. Second, compared with other antidiabetic drugs, these agents are often prescribed to patients with high cardiovascular risk, chronic kidney disease, or obesity, making it challenging to adequately address confounding by indication, although an active comparator new-user study design may help alleviate this issue. 132 Comparing SGLT2 inhibitors to GLP-1 receptor agonists could be an option, as these drugs are sometimes considered alternatives. 11

In addition to clinical research on VTE, fundamental, preclinical research, as well as clinical research on intermediate endpoints, should be encouraged to explore potential effects—either beneficial or harmful—on thrombosis and hemostasis, where currently available evidence is also scarce. An animal study by Chien et al. 133 demonstrated that treatment with exendin-4 (a GLP-1 receptor agonist) restored normal endothelial morphology and improved arteriovenous fistula function in rats with chronic kidney disease. Steven et al. 134 found that GLP-1 receptor activation in platelets by linagliptin (a DPP-4 inhibitor) and liraglutide (a GLP-1 receptor agonist) significantly attenuated endotoxemia-induced microvascular thrombosis. An animal study by Evlakhov et al. 135 showed that pretreatment with dapagliflozin (an SGLT2 inhibitor) reduced endothelial permeability under conditions of PE. An RCT conducted among obese, non-diabetic/prediabetic patients undergoing hip surgery (receiving dabigatran for anticoagulation postsurgery) investigated the effects of liraglutide versus placebo on coagulation activation. 136 No changes were observed in coagulation activation, but factor VIII levels were significantly higher 2 hours postsurgery, while D-dimer levels were significantly lower 3 days postsurgery. Another RCT involving overweight and/or insulin-resistant women with polycystic ovary syndrome who received liraglutide or placebo found no effect on endogenous thrombin potential, though there was a trend toward decreased plasminogen activator inhibitor-1. 137 These studies might provide valuable insights into the potential mechanisms and effects of these novel medications on VTE.

It is important to note that all the studies reviewed focused exclusively on the risk of primary VTE. Thus, a knowledge gap remains regarding whether these novel antidiabetic drugs affect the risk of VTE recurrence in a population with a history of VTE, as well as the efficacy or safety of anticoagulants used for preventing VTE recurrence. Given the moderately high absolute risk of VTE recurrence, 138 from a statistical power perspective, it may be more feasible to conduct studies on VTE recurrence. There is also limited knowledge about potential interactions between anticoagulants and novel antithrombotic agents, with most of the available evidence pertaining to warfarin in healthy individuals. 139 140 141 142 These areas represent important future research directions.

## Conclusion

In conclusion, the current evidence regarding the potential effects of novel antidiabetic drugs on VTE risk is very limited and inconsistent. Further studies are warranted to bridge this knowledge gap and guide clinical decision-making.
