# Supplementary material for: Novel Antidiabetic Drugs and Risk of Venous Thromboembolism: A Literature Review
Source: Semin Thromb Hemost. 2025 Mar 28;51(7):756–67. doi: 10.1055/a-2546-0353 (PMC12431824; doi:10.1055/a-2546-0353)
Supplement: Supplementary file 1 — Supplementary Material [file 10-1055-a-2546-0353-s250061ra.pdf]

Appendix A1

PubMed Search Strategy

#1 Glucagon-like peptide-1 (GLP-1) analogs
"Glucagon-Like Peptide 1"[Mesh] OR "Liraglutide"[Mesh] OR "Glucagon-Like Peptide 1"[TIAB] OR "Glucagon Like Peptide 1"[TIAB] OR "GLP-1"[TIAB] OR "GLP 1"[TIAB] OR "Glucagon-Like Peptide-1"[TIAB] OR "exenatide"[TIAB] OR "liraglutide"[-TIAB] OR "lixisenatide"[TIAB] OR "albiglutide"[TIAB] OR "dulaglutide"[TIAB] OR "semaglutide"[TIAB] OR "beinaglutide"[-TIAB] OR "Victoza"[TIAB] OR "Saxenda"[TIAB] OR "NN 2211"[TIAB] OR "2211, NN"[TIAB] OR "NN2211"[TIAB] OR "NN-2211"[TIAB] OR "Glucagon-like peptide-1 analogues"[TIAB] OR "GLP-1 analogues"[TIAB]

#2 Dipeptidyl peptidase 4 (DPP-4) inhibitors
"Dipeptidyl-Peptidase IV Inhibitors"[Mesh] OR "Dipeptidyl-Peptidase IV Inhibitors"[TIAB] OR "Dipeptidyl Peptidase IV Inhibitors"[TIAB] OR "DPP-4 Inhibitor"[TIAB] OR "DPP 4 Inhibitor"[TIAB] OR "Inhibitor, DPP-4"[TIAB] OR "DPP-IV Inhibitor"[TIAB] OR "DPP IV Inhibitor"[TIAB] OR "Inhibitor, DPP-IV"[TIAB] OR "DPP-4 Inhibitors"[TIAB] OR "DPP 4 Inhibitors"[TIAB] OR "DPP-IV Inhibitors"[TIAB] OR "DPP IV Inhibitors"[TIAB] OR "Gliptin"[TIAB] OR "Dipeptidyl Peptidase 4 Inhibitor"[TIAB] OR "Dipeptidyl-Peptidase IV Inhibitor"[TIAB] OR "Dipeptidyl Peptidase IV Inhibitor"[TIAB] OR "Dipeptidyl-Peptidase 4 Inhibitor"[TIAB] OR "Inhibitor, Dipeptidyl-Peptidase 4"[TIAB] OR "Dipeptidyl-Peptidase 4 Inhibitors"[TIAB] OR "Dipeptidyl Peptidase 4 Inhibitors"[TIAB] OR "Gliptins"[TIAB] OR "DPP4 Inhibitor"[TIAB] OR "Inhibitor, DPP4"[TIAB] OR "DPP4 Inhibitors"[TIAB] OR "Dipeptidyl peptidase 4 inhibitors"[TIAB] OR "DPP-4 inhibitors"[TIAB] OR "sitagliptin"[TIAB] OR "vildagliptin"[TIAB] OR "saxagliptin"[TIAB] OR "alogliptin"[TIAB] OR "linagliptin"[TIAB] OR "gemigliptin"[TIAB] OR "evogliptin"[TIAB] OR "teneligliptin"[TIAB]

#3 Sodium-glucose co-transporter 2 (SGLT2) inhibitors
"Sodium-Glucose Transporter 2 Inhibitors"[Mesh] OR "Sodium Glucose Transporter 2 Inhibitors"[TIAB] OR "Sodium-glucose cotransporter protein-2 inhibitors"[TIAB] OR "SGLT-2 Inhibitors"[TIAB] OR "SGLT 2 Inhibitors"[TIAB] OR "SGLT2 Inhibitors"[TIAB] OR "Sodium-Glucose Transporter 2 Inhibitor"[TIAB] OR "Sodium Glucose Transporter 2 Inhibitor"[TIAB] OR "SGLT2 Inhibitor"[TIAB] OR "Inhibitor, SGLT2"[TIAB] OR "Gliflozins"[TIAB] OR "Gliflozin"[TIAB] OR "SGLT-2 Inhibitor"[TIAB] OR "Inhibitor, SGLT-2"[TIAB] OR "SGLT 2 Inhibitor"[TIAB] OR "Sodium-glucose co-transporter 2 inhibitors"[TIAB] OR "dapagliflozin"[TIAB] OR "canagliflozin"[TIAB] OR "empagliflozin"[TIAB] OR "ertugliflozin"[TIAB] OR "ipragliflozin"[TIAB] OR "sotagliflozin"[TIAB] OR "luseogliflozin"[TIAB] OR "Remogliflozin"[TIAB] OR "Sergliflozin"[TIAB] OR "Sotagliflozin"[TIAB] OR "bexagliflozin"[TIAB]

#4 Venous thromboembolism
"Venous Thrombosis"[Mesh] OR "Venous Thrombosis"[tiab] OR "Thrombosis, Venous"[tiab] OR "Thromboses, Venous"[-tiab] OR "Venous Thromboses"[tiab] OR "Phlebothrombosis"[tiab] OR "Phlebothromboses"[tiab] OR "Deep Vein Thrombosis"[tiab] OR "Deep Vein Thromboses"[tiab] OR "Thromboses, Deep Vein"[tiab] OR "Vein Thromboses, Deep"[tiab] OR "Vein Thrombosis, Deep"[tiab] OR "Thrombosis, Deep Vein"[tiab] OR "Deep Venous Thrombosis"[tiab] OR "Deep Venous Thromboses"[tiab] OR "Thromboses, Deep Venous"[tiab] OR "Thrombosis, Deep Venous"[tiab] OR "Venous Thromboses, Deep"[tiab] OR "Venous Thrombosis, Deep"[tiab] OR "Deep-Vein Thrombosis"[tiab] OR "Deep-Vein Thromboses"[tiab] OR "Thromboses, Deep-Vein"[tiab] OR "Thrombosis, Deep-Vein"[tiab] OR "Deep-Venous Thrombosis"[tiab] OR "Deep-Venous Thromboses"[tiab] OR "Thromboses, Deep-Venous"[tiab] OR "Thrombosis, Deep-Venous"[tiab] OR "Venous Thromboembolism"[Mesh] OR "Venous Thromboembolism"[tiab] OR "Thromboembolism, Venous"[tiab] OR "Pulmonary Embolism"[-Mesh] OR "Pulmonary Embolism"[tiab] OR "Pulmonary Embolisms"[tiab] OR "Embolism, Pulmonary"[tiab] OR "Embolisms, Pulmonary"[tiab] OR "Pulmonary Thromboembolisms"[tiab] OR "Pulmonary Thromboembolism"[tiab] OR "Thromboembolism, Pulmonary"[tiab] OR "Thromboembolisms, Pulmonary"[tiab]

#5 (#1 OR #2 OR #3) AND #4
The search was conducted on February 5, 2025, and yielded 46 results.

 © 2025. Thieme. All rights reserved.
